# The Role of the Gut Microbiome in Cancer Immunotherapy: Current Knowledge and Future Directions

**DOI:** 10.3390/cancers15072101

**Published:** 2023-03-31

**Authors:** Despoina E. Kiousi, Antonia Z. Kouroutzidou, Konstantinos Neanidis, Emmanuel Karavanis, Dimitrios Matthaios, Aglaia Pappa, Alex Galanis

**Affiliations:** 1 Department of Molecular Biology and Genetics, Faculty of Health Sciences, Democritus University of Thrace, 68100 Alexandroupolis, Greece; 2 Oncology Department, 424 General Military Training Hospital, 56429 Thessaloniki, Greece; 3 Oncology Department, Rhodes General Hospital, 85133 Rhodes, Greece

**Keywords:** gut microbiota, immunotherapy, immune checkpoint inhibitors, probiotics, fecal microbiota transplantation, tumor microenvironment

## Abstract

**Simple Summary:**

Cancer immunotherapy is a treatment modality that involves the stimulation of the patient’s immune system to fight off tumors. Although efficient in limiting the disease progression of several solid tumors, including lung cancer and melanoma, some patients may have poor outcomes. This review focuses on the role of the gut microbiota (the microbial community residing in the gastrointestinal tract) in immunity and cancer immunotherapy. Manipulation of the gut microbiota with dietary interventions or fecal microbiota transplantation to enhance response to immunotherapy could pave the way for personalized therapies with improved efficacy.

**Abstract:**

Cancer immunotherapy is a treatment modality that aims to stimulate the anti-tumor immunity of the host to elicit favorable clinical outcomes. Immune checkpoint inhibitors (ICIs) gained traction due to the lasting effects and better tolerance in patients carrying solid tumors in comparison to conventional treatment. However, a significant portion of patients may present primary or acquired resistance (non-responders), and thus, they may have limited therapeutic outcomes. Resistance to ICIs can be derived from host-related, tumor-intrinsic, or environmental factors. Recent studies suggest a correlation of gut microbiota with resistance and response to immunotherapy as well as with the incidence of adverse events. Currently, preclinical and clinical studies aim to elucidate the unique microbial signatures related to ICI response and anti-tumor immunity, employing metagenomics and/or multi-omics. Decoding this complex relationship can provide the basis for manipulating the malleable structure of the gut microbiota to enhance therapeutic success. Here, we delve into the factors affecting resistance to ICIs, focusing on the intricate gut microbiome–immunity interplay. Additionally, we review clinical studies and discuss future trends and directions in this promising field.

## 1. Introduction

Immunotherapy dramatically changed the course of cancer treatment and revitalized the field of tumor immunology. Immunotherapy regimens recruit the immune system of the patient to fight off cancer, representing a standing example of precision medicine. To date, adoptive cell transfer, oncolytic virus therapies, cancer vaccines, cytokine therapies, immune checkpoint inhibitors, and divalent antibodies have gained traction. Among them, ICIs are proving to be a promising treatment option for cancer patients due to their long-lasting effects and improved overall survival and tolerance compared to conventional treatment [1,2]. ICIs are monoclonal antibodies designed to enhance the T cell-mediated anti-tumor immune response by blocking inhibitory receptors and ligands [3]. Ipilimumab, a human cytotoxic T-lymphocyte antigen 4 (CTLA-4)-blocking antibody indicated for the treatment of advanced melanoma, was the first ICI that received approval, opening a new era in cancer immunotherapy [4]. Almost immediately after, monoclonal antibodies targeting the programmed cell death protein 1 (PD-1) (pembrolizumab and nivolumab) and its ligand, PD-L1 (atezolizumab and durvalumab), obtained FDA approval; today, they are the most widely prescribed monoclonal antibodies due to their remarkable clinical efficacy [5]. The award of the Nobel Prize in Physiology or Medicine in 2018 to Tasuku Honjo and James Allison for their discovery of cancer therapy via inhibition of negative immune regulation was a milestone in the field of immuno-oncology. Currently, hundreds of patients are prescribed with ICIs both in the first line and later lines of treatment, alone or in combination with chemotherapy or targeted therapy [6,7]. However, although generally efficient in limiting the progression of the disease or even giving partial or complete response to some patients, a significant proportion of patients exhibit innate or acquired resistance to ICIs (non-responders), which is attributed to either host-related factors or resistance after initial response [8]. Some factors that may influence the response to ICIs are host- or tumor-related, including the antigenicity of the tumor cells and tumor microenvironment (TME) as well the gut microbiome.

The human gut microbiome is currently a topic of intense research due to its significant contribution to health and disease. Alterations in the structure and metabolic capacity of the intestinal microbiota are repeatedly associated with susceptibility to various immune-related pathological conditions, including inflammatory bowel disease, autoimmune disorders, chronic inflammation, and cancer [9]. In the last few years, studies suggested the correlation of the gut microbiome to the efficacy and therapeutic toxicity of ICI immunotherapy. The first evidence came from the breakthrough preclinical study by Sivan et al. that highlighted the correlation of gut commensal populations of *Bifidobacterium* with delayed tumor growth and enhanced T cell tumor infiltration and anti-tumor immunity, supporting the efficacy of PD-L1 blockade [10]. Several preclinical and clinical studies have been published since, trying to establish a causative relationship between microbial signatures and response to ICI treatment [11,12,13]. Concomitantly, interventional studies aimed to design strategies for the manipulation of gut microbiota composition to maximize effect and minimize adverse events in patients with solid carcinomas receiving ICIs. In this review article, we (i) describe anti-tumor immunity, ICI resistance, and the highly dynamic and complex microbiome–immune interplay, (ii) summarize recent clinical data on the effect of gut microbiota on ICIs response, and (iii) identify future trends, pitfalls, and opportunities in the field by focusing on ongoing clinical trials.

## 2. Anti-Tumor Immune Response, Immunological Tolerance, and Resistance to ICI Therapy

The concept of cancer immunosurveillance was first proposed by Dr. Ehrlich in 1909 and refers to the hypothesis that the immune system lies in wait for the appearance of transformed cancer cells that it can immediately eliminate before they develop into malignant tumors [14]. This hypothesis is supported by the increased incidence of tumor growth in immunodeficient mice and humans [15]. The immune response to cancer cells involves a repeating sequence of steps, known as the cancer immunity cycle, that can be summarized in three phases: elimination, equilibrium, and escape [16]. At early stages of elimination, the enhanced production of neo-antigens by cancer cells, as an inevitable result of their enhanced genetic instability [17], leads to their capture by antigen-presenting cells, specifically dendritic cells (DCs), which leave the tumor microenvironment and migrate to the lymph nodes, inducing the activation of tumor-specific and cytotoxic CD8+ T cells. These cells migrate to tumors, where they can recognize and eliminate cancer cells. In this cytotoxic environment, neo-antigens are constantly released, fueling cancer cell clearance [18]. During the elimination phase, the balance is tipped towards anti-tumor immunity [19] due to the increased expression of neo-antigens in combination with the high frequency of major histocompatibility complex (MHC) class I molecules on the surface of tumor cells and the existence of apoptosis-inducing agents (perforin, granzymes, Fas, and tumor necrosis factor-related apoptosis-inducing ligand (TRAIL) receptors) as well as anti-tumor factors, interferon (IFN)-α/β/γ, interleukin (IL)-1, IL-12, and tumor necrosis factor-alpha (TNF-α). In this way, the immune system successfully locates and eliminates most of the transformed cells, rejecting any potential tumors. Despite immunosurveillance and elimination of cancer cells, carcinogenesis may proceed regardless [19]. Indeed, constant immune pressure leads cancer cells to develop resistance to both innate and adaptive anti-tumor responses and to gradually accumulate a multitude of genetic and epigenetic modifications that alter their properties and shield them against immune recognition (equilibrium phase). In this dormant stage, a balance between anti-tumor (IL-12 and IFN-γ) and tumor-promoting cytokines (IL-10 and IL-23) is established. The loss of tumor antigens and MHC class I molecules from the cancer cell surface is usually an important trigger for the transition to the last stage, escape, when the immune system fails to limit tumor growth, resulting in its appearance as a clinical disease [20].

Immune checkpoint factors limit aberrant immune responses during infection, autoimmune disease, and cancer, thereby sustaining immunological homeostasis [21]. However, the very same molecules play a pivotal role in the immune escape of cancer cells. The most studied immune checkpoint factors are PD-1 (also known as CD279), its ligand PD-L1, and CTLA-4. These molecules can inhibit T cell activation and proliferation at late and early stages, respectively. More specifically, PD-1 is expressed on activated T cells, whereas PD-L1 and PD-L2 are presented on tumor cells or other cells found in the TME, including DCs and natural killer (NK) cells. Receptor–ligand interactions lead to antigen-specific T cell apoptosis and the inhibition of regulatory T cell (Tregs) apoptosis [22]. These events cause a decrease in T cell-mediated anti-tumor immunity and the production of IFN-γ, TNF-α, and IL-2, and it enhances the proliferation of DCs [23]. Similarly, CTLA-4 is expressed in T cells at an earlier stage, presenting high affinity to CD80 and CD86, thereby depriving lymphocytes of co-stimulatory signals [24]. Furthermore, experimental data suggest the expression of CTLA-4 in B cells, natural killer T cells (NKT) and NK cells, and DCs; however, its role in these cell populations is not clearly established [25]. Nonetheless, CTL-4 contributes to the dampening of the immune response by decreasing the production of IL-2 and inhibiting T-cell activation. Soluble forms of both receptors and ligands were found in the plasma of patients; however, their mode of action is still unclear. Some may halt inhibitory interactions and promote anti-tumor immunity, whereas others may further promote immune escape [21]. Immune checkpoint blockade is based on limiting the PD-1/PD-L1 or PD-L2 and CTLA-4/CD80 or CD86 interactions, thereby diminishing these phenomena. It is suggested that anti-CTLA-4 may be considered a more effective treatment regimen due to its interference with the initial steps of T cell activation, whereas anti-PD-L1 therapy specifically targets tumor-specific T cell populations [26]. Clinical data suggest that anti-PD-L1 treatment may elicit a more sustained response to patients with melanoma compared to CTLA-4 blockade [27]. Additionally, anti-CTLA-4 antibodies are linked to a higher incidence of immune-related adverse effects (irAEs) compared to anti-PD-L1 antibodies, possibly due to their interference with early stages of T cell development [28]. Nonetheless, ICIs are effectively used as a monotherapy or combinatory therapy in the clinical practice of newly diagnosed patients or patients that have previously received conventional anti-tumor modalities [21].

ICI therapy shows improved results against specific types of tumors, including non-small cell lung cancer (NSCLC) and advanced head and neck squamous cell carcinoma (HNSCC) compared to conventional treatment [3], even promoting sustained outcomes and long-term survival in patients with advanced melanoma [29]. However, a significant proportion of patients may develop primary or acquired resistance. The immune context of the host (e.g., T cell activity and repertoire of T-cell receptors) and tumor antigenicity (including mutational burden) are important determinants of the response to ICIs [30]. More specifically, anti-tumor responses are largely dependent on T cell activity and T cells repertoire built from a young age as well as on the priming of pro-inflammatory response and overall immunocompetence [26]. A high tumor mutational burden leads to higher antigenicity and immunological recognition, and thus, it leads to higher immunological clearance [26]. For example, mutations in key genes coding for Janus kinases (JAK) and/or for the phosphatase and tensin homolog deleted on chromosome 10 (PTEN) or genes involved in the human leukocyte antigen (HLA) class I complex and IFN-γ pathways may promote immunological resistance and escape [31]. Lymphocyte tumor infiltration depends on TME architecture and immunomodulatory signaling molecules present in situ. Based on the presence and positioning of immunological populations in the TME, tumors can be described as “hot”, altered (excluded or inflamed), or “cold”. “Cold” tumors are immune deserts, whereas “hot” tumors are highly infiltrated with effector T cells, presenting a pro-inflammatory phenotype [32,33]. Altered tumors present lower immunogenicity than ‘’hot’’ tumors, as lymphocytes at the periphery are unable to penetrate the TME due to immunosuppressive conditions in situ or structural changes that prevent infiltration [29]. In this context, the heightened presence of specific DC populations with immunosuppressive activity (e.g., myeloid-derived suppressor cells and regulatory DCs) or tumor-associated macrophages (TAMs) is related to poor prognoses [31]. Overall, patients with altered or hot tumors have better outcomes. Despite initial responses to treatment, a proportion of patients relapses due to the development of acquired resistance to ICIs, which can be attributed to a plethora of mechanisms, including T cell exhaustion, loss of neoantigen presentation, and mutation variants that enhance immunological escape [34]. Conclusively, at the core of ICI resistance lies the capacity of the immune system to locate and eliminate tumor cells, which is determined by host-specific factors (including genetics and environmental conditions) and the ability of cancer cells to evade immunological recognition and clearance.

## 3. The Gut Microbiota–Immune System Crosstalk and Anti-Tumor Immunity

The gut is colonized by a vast number of microorganisms, including bacteria, fungi, and viruses, which collectively make up the gut microbiome. These microorganisms create a complex micro-ecosystem that co-evolved with the host. The gut microbiota not only contribute to the digestion and fermentation of food, but it can also affect the function of the intestinal immune system, regulating both innate and adaptive immune responses [9]. The intimate relationship between the microbiome and the immune system of the host is built from birth, as studies show that it is responsible for the maturation of mononuclear populations [9]. The immune system–microbiota crosstalk is mediated by both surface molecules (Microbe Associated Molecular Patterns, MAMPs) and excreted metabolic byproducts. More specifically, pattern recognition receptors (PRRs, mainly toll-like receptors (TLRs), anchored on the cell or endosome membranes of intestinal epithelial and innate immune cells participate in the detection of these compounds. Antigen recognition by TLRs results in the modulation of local immune responses, gut barrier integrity, and the overall homeostasis of the host [35]. Surface molecules that signal through these receptors are peptidoglycan and lipopolysaccharide (LPS), the main components of the cell wall and outer membranes of gram-positive and gram-negative microorganisms, or other more specialized antigens produced by gut commensals. For example, polysaccharide A (PSA), which is produced by *Bacteroides fragilis*, plays a significant role in immune system education and modulation by enhancing the inhibitory activity of human CD39+Foxp3+ T cells [36,37]. Other commensals may act through the activation of the NOD-like receptors (NLR) family pyrin domain containing 3 (NRLP3) inflammasome or absent in melanoma 2 (AIM2) inflammasome signaling, whereas some may stimulate the production of IgA in the gut or the activity of local Th17 populations, as reviewed in great length by Zheng et al. [9]. Antigen sampling in the gut is performed by several cell populations including goblet cells, intestinal M cells residing on the overlying epithelium of Peyer patches (lymphoid follicles of the mucus membrane of the small intestine), and lymph nodes. Furthermore, underlying immunological populations that can sample the gut lumen content using transepithelial projections (macrophages and DCs) participate in this phenomenon. The activity of these cell populations leads to the stimulation of mononuclear phagocytes of the lamina propria and the subsequent modulation of adaptive immunity [38]. Furthermore, microbe sampling through M cells was shown to be fundamental for the establishment of soluble IgA in the small intestine during early life, ensuring gut homeostasis [39].

Additionally, gut microbiota-derived metabolites produced through the anaerobic fermentation of food residue in the digestive tract, most abundantly short-chain fatty acids (SCFAs: acetate, propionate, and butyrate), diffuse through the intestinal epithelium and reach the systemic circulation, exerting important immunomodulatory effects [40]. Butyrate was shown to suppress LPS-induced maturation and metabolic reprogramming of human monocyte-derived DCs, conditioning them to polarize naive CD4+ T cells toward IL-10-producing type 1 regulatory T cells [41]. In the TME, gut microbiota metabolites including SCFAs, deoxycholic or petrocholic acids, and inosine can locally affect either the inflammatory landscape [42] or angiogenesis and metastatic potential [43,44]. In this context, the production of butyrate by *Faecalibacterium prausnitzii* limited angiogenesis in vitro by downregulating the expression of hypoxia-inducible factor 1-alpha (HIF-1α) and subsequently that of vascular endothelial growth factor (VEGF) [44]. It is suggested that small molecules derived from gut commensals can influence T cell repertoire and reactivity. For example, the colonization of germ-free mice with a defined microbial consortium (altered Schoedler flora) resulted in the expansion of colonic Treg cells, leaving unaffected populations in the spleen or mesenteric lymph nodes. This effect was presumably mediated via the stimulation of TLR-signaling from soluble microbiota-derived compounds [45]. Segmented filamentous bacteria (SFB) can trigger the production of pro-inflammatory mediators in the gut, leading to the lowering of T cell activation thresholds. In this context, SFB led to the prolonged activation of Th1 cells in the gut and enhanced recognition of endogenous antigens while bypassing the mucosal tolerance mechanisms [46]. The existence of homology between the non-self-gut microbiota epitopes and tumor antigens could result in the stimulation of T cells and the initiation of anti-tumor immunity [24]. Based on this hypothesis, central memory T cells can be released in the systemic circulation and accumulate in the tumor bed, in which they differentiate into effector T cells that may target and eliminate tumor cells [47]. Overall, the ability of gut commensals to fine tune local and systemic immune responses indicates that the unique composition of the gut microbiota of patients may promote or limit the efficacy of treatment targeting anti-tumor immunity, thereby enhancing or limiting treatment response, tumor clearance, and survival.

## 4. The Gut Microbiome and Modulation of ICI Response

The ability of gut microbiota to modulate anti-tumor immunity suggests a possible implication for immunotherapy success. In recent years, the number of studies investigating this relationship grew exponentially, focusing mainly on late-stage melanoma and lung cancer, as shown in Table 1. Toward this direction, Frankel et al. were the first to correlate gut microbiome composition with response to ICIs in patients with metastatic melanoma using shotgun metagenomics. *Bacteroides caccae* abundance at baseline was related to elevated responses to ICI immunotherapy, irrespective of the checkpoint inhibitor blockade used [48]. Furthermore, gut metabolomic profiling showed that the response to ICIs was positively associated with anacardic acid, which may exert anti-tumor effects by stimulating T cell recruitment in situ [48]. Similar to this, a high abundance of *B. thetaiotamicron* was correlated with better response to ICIs therapy and *B. massiliensis*, with prolonged progression-free survival (PFS) in patients with unresectable metastatic melanoma [49]. However, other studies reported that metastatic melanoma patients receiving anti-CTLA-4 treatment with baseline gut microbiota enriched with *Bacteroides* showed lower response rates [50], and similarly, patients with a high prevalence of *B. thetaiotamicron* and *E. coli* were less responsive to anti-PD-1 therapy [12]. The conflicting data on the effect of members of the *Bacteroides* genus on ICI efficiency underlines the possibility that favorable effects on response to ICIs may be elicited on a species-specific basis while also being influenced by host factors [11]. Several clinical studies suggest the correlation between members of the *Faecalibacterium* genus or the Firmicutes phylum with elevated responses to anti-PD-1 [12] or anti-CTLA-4 immunotherapy [50], and higher survival rates of melanoma patients [51], as their prevalence is positively correlated with higher T cell infiltration in the TME and higher counts of CD4+ and CD8+ T cells at the periphery [48]. Furthermore, a positive association of bifidobacteria populations with response to immunotherapy in melanoma patients was indicated in a recent study [52]. To this end, preclinical studies revealed that bifidobacterial populations are correlated with an increased accumulation of antigen specific CD8+ T cells within the TME and with an elevated expression of genes associated with antitumor immunity in DCs, supporting T cell activation [10]. In this sense, a previous study on the immunomodulatory effects of bifidobacteria showed that the minimization of their intestinal population using antibiotics in neonatal rats led to a significant delay in the maturation of DCs in Peyer’s patches and development of T cells in the thymus. Subsequently, a decreased IFN-γ/IL-4 ratio and expression of IL-10 and IL-12 were recorded in intestinal mucosa and cultured peripheral blood mononuclear cells (PBMCs) as well as a lower expression of IgM in cultured PBMCs. A disrupted balance of Th1 and Th2 cells was subsequently reported. The external administration of *Bifidobacterium* spp. reversed this phenotype [53]. *B. pseudolongum* administration combined with anti-CTLA-4 immunotherapy was found to induce Th1 differentiation and effector T cell function, which was supported by elevated IFN-γ production in splenic CD4+ and CD8+ T lymphocytes in tumor-free mice. By delving into the molecular mechanism behind these findings, it was shown that intestinal *B. pseudolongum* induced these effects through the production of inosine, a key bacterial-derived metabolite, acting through T cell-specific adenosine A2A receptor (A2AR) signaling. More specifically, inosine is required for the sufficient co-stimulation of T cells (likely by DCs), IL-12 receptor engagement for Th1 differentiation, and IFN-γ production [42]. IFN-γ promotes cell death by inducing the expression of pro-apoptotic proteins, modulates cellular immunity, and orchestrates anti-tumor immune responses via the stimulation of cytotoxic T cell populations [54]. It is therefore a key player in response to immunotherapy. The increased expression of interferon signature genes CD274, lymphocyte-activation gene 3 (LAG3), and chemokine (C-X-C motif) ligand 9 (CXCL9) or higher IFN-γ concentration in the TME is linked to better ICI efficacy [55].

Immunotherapy, mainly PD-1 blockade, is a commonly employed strategy for advanced lung cancer treatment due to the heightened response of its patients compared to chemotherapy [56]. Similar to findings from studies conducted on melanoma, distinct bacterial populations are correlated with response to treatment in lung cancer patients receiving ICIs (Table 1). In one study, patients harboring gut microbiota enriched with bacteria related to pro-inflammatory outcomes, including the gram-positive *B. longum* and gram-negative LPS-producing bacteria *Akkermansia muciniphila*, *Alistipes*, and *Porphyromonas*, exhibited better clinical responses to immunotherapy. In the context of advanced thoracic carcinoma, a higher abundance of Akkermansiaceae, Enterococcaceae, Enterobacteriaceae, Carnobacteriaceae, and Clostridiales Family XI in feces at the time of diagnosis was associated with better responses and longer PFS [57]. Moreover, SCFA producers (*Faecalibacterium prausnitzii*, *A. muciniphila*, *Bifidobacterium* spp., *Lactobacillus* spp., and *Streptococcus* spp.) were found in increased abundance in responder groups of clinical trials [58,59], being positively correlated with anti-PD-1/PD-L1 treatment responses in patients with lung [60], gastrointestinal [61], and hepatocellular cancer [62] or melanoma [63]. Among the effector strains, *A. muciniphila* was found to utilize inosine-A2AR signaling for its ICI-promoting effect, which is similar to bifidobacteria [58,64]. Mechanistic studies in a mouse model of prostate cancer showed that this gut commensal can induce the numbers of M1-like macrophages and IFNγ+CD8+ T cells [65] and that its membrane phospholipids can trigger the production of a subset of pro-inflammatory cytokines through interactions with the TLR2–TLR1 complex [66]. Concomitantly, *A. muciniphila* may elicit favorable effects in the context of immunotherapy by suppressing the functionality of Tregs [67,68]. Furthermore, high lactobacilli abundance was correlated with prolonged time to treatment failure in NSCLC patients treated with ICIs in a small-scale clinical trial [69]. Lactobacilli can induce strain-specific immunomodulatory effects on the host. For example, using a syngeneic subcutaneous CT26 tumor mouse model, it was shown that *Lc. casei* ATCC 393 administration increased T cell tumor infiltration and production of Th1 immunostimulatory cytokines, which subsequently led to impaired tumor growth [70]. Towards this direction, previous studies from our lab showed that local administration of *Lp. pentosus* B281 and *Lp. plantarum* B282 [71] or of *Lc. paracasei* K5 [72] in a dorsal-air-pouch mouse model of inflammation induced rapid production of pro-inflammatory cytokines and enhanced T cell infiltration.

**Table 1:** Clinical trials investigating the effect of gut microbiota composition on ICI immunotherapy efficacy and therapeutic toxicity.

Participants	Disease Stage	Immuno-Therapy type	Study Design	Samples	Analysis	Findings	Microbiota Diversity	Ref.
**Melanoma**								
Unresectable/ metastatic melanoma (*n* = 39, 30M/9F)	IV	Anti-PD-1 or anti-CTLA-4, or anti-PD-1/anti-CTLA-4	Assessment of GM composition at baseline and before each ICIs infusion	Feces	MSS, UPLC-MS/MS	↑*B. caccae*, *F. prausnitzii*, *B. thetaiotamicron*, and *Holdemania filiformis, D. formicogenerans* in R ↑Anacardic acid in R	No significant differences between R and NR	[48]
Unresectable cutaneous melanoma (*n* = 25, 10M/15F)	IIIc/IV	Anti-PD-1, or anti-PD-1/anti-CTLA-4	Assessment of overall gut microbiome composition, relative microbial abundance, and differences in prevalence between responders and non-responders	Feces	MSS	↑*Ruminococcus gnavus*, *E. coli*, *E. biforme*, *Phascolarctobacterium succinatutens*, and *Streptococcus salivarius* in R ↑*B. longum*, *Prevotella copri*, *Coprococcus* sp ART55-1, *Eggerthella* unclassified, and *Eubacterium ramulus* in NR ↑*Streptococcus parasanguinis* carriers → longer OS ↑*B. massiliensis* → longer PFS ↑*Peptostreptococcaceae* (unclassified species) carriers → shorter OS and PFS 17 microbial pathways differentially enriched between R and NR	No significant differences between R and NR	[49]
Metastatic melanoma (*n* = 26, 13M/13F)	IV	CTLA-4 blockade	Assessment of GM composition and blood-based biomarkers at baseline, before each ipilimumab infusion, at the end of the treatment, and at the time of colitis	Feces, Blood serum	16S rRNA gene sequencing, Immunopheno-typing, Soluble immune markers analysis	Baseline GM enriched with *Faecalibacterium* spp. and other Firmicutes → longer PFS and OS Baseline GM enriched with *Bacteroides* spp. → No ipilimumab-induced colitis	No significant differences	[50]
Metastatic melanoma (*n* = 112)	IV	PD-1 blockade	Assessment of oral and gut microbiome composition at baseline	Buccal swabs, Feces, Tumor biopsies, Blood	16S rRNA gene sequencing	↑Clostridiales/Ruminococcaceae and *Faecalibacterium* spp. in R ↑Bacteroidales in NR ↑*Faecalibacterium* abundance → prolonged PFS	↑α-diversity in R → prolonged PFS	[12]
Metastatic melanoma (*n* = 27, 21M/6F)	III (*n* = 9) IV (*n* = 18)	Anti-PD-1/ anti-CTLA-4	Assessment of gut microbiome overall diversity and composition Correlation with PFS	Feces	16S rRNA gene sequencing, MSS	↑*F. prausnitzii*, *Coprococcus eutactus*, *Prevotella stercorea*, *Streptococcus* spp., and *Lachnospiraceae bacterium* → longer PFS ↑*Bacteroides* spp., *Ruminococcus* *gnavus*, and *Blautia producta* abundance → shorter PFS	Higher community diversity → longer PFS	[55]
Metastatic melanoma (*n* = 42, 20M/22F)	IV	Anti-PD-1/ anti-CTLA-4	Assessment of GM composition before treatment	Feces	16S rRNA gene sequencing, MSS	↑*E. faecium, Collinsella aerofaciens, B. adolescentis, Klebsiella pneumoniae, Veillonella parvula, Parabacteroides merdae, Lactobacillus* sp*.,* and *B. longum* in R ↑*Ruminococcus obeum* and *Roseburia intestinalis* in NR	ND	[63]
**Non-small cell lung cancer**								
NSCLC (*n* = 11, 8M/3F)	IV	PD-1 blockade	Assessment of gut microbiota composition at baseline and during immunotherapy Comparison between patients and healthy controls	Feces	16S rRNA sequencing, Meta-metabolomics (GC–MS/SPME)	↑*A. muciniphila*, *B. longum, Faecalibacterium prausnitzii* in R ↑*Propionibacterium acnes, Veillonella, Staphylococcus aureus, Peptostreptococcus, Ruminococcus bromii*, *Dialister*, and *Sutterella* in NR ↑*Rikenellaceae*, *Prevotella*, *Streptococcus*, *Lactobacillus*, *Bacteroides plebeius*, *Oscillospira*, and Enterobacteriaceae enriched in patients compared to HC	ND	[58]
Advanced ΝSCLC (*n* = 37, 29M/8F)	IIIB (*n* = 6) IV (*n* = 31)	PD-1 blockade	Assessment of gut microbiota composition at baseline and prior to infusion	Feces, Blood	16S rRNA sequencing, Flow cytometry	↑Μicrobiome diversity → Better response, prolonged PSF ↑*Alistipes putredini*, *Prevotella copri*, *B. longum*, *Lachnobacterium* sp, Lachnospiraceae, and *Shigella* in R ↑*Ruminococcus_unclassified* in NR	α-diversity; significantly higher in R vs. NR at baseline β-diversity; significant difference between R and NR	[59]
Advanced NSCLC (*n* = 17, 13M/4F)	III (*n* = 6) IV (*n* = 8) POR (*n* = 3)	PD-1 blockade	Assessment of gut microbiota composition during treatment along with clinical evaluations and response to immunotherapy	Feces	16S rRNA sequencing	↑*Lactobacillus* and *Clostridium* in patients with longer TTF ↓*Bilophila* and *Sutterella* in patients with prolonged TTF ↑*Lactobacillus*, *Clostridium*, and *Syntrophococcus* in R ↑*Bilophila*, *Sutterella*, and *Parabacteroides* in NR	α-diversity; No significant differences between R and NR	[69]
NSCLC (*n* = 63, 53M/10F)	III (*n* = 10) IV (*n* = 53)	PD-1 blockade	Assessment of overall gut microbiome composition prior to immunotherapy	Feces	MSS	↑*Methanobrevibacter* and *Parabacteroides* in patients PFS ≥ 6 months ↑*Veillonella*, *Selenomonadales*, and *Negativicutes* in patients with PFS < 6 months	β-diversity; significant differences between patients with PFS ≥ 6 months and patients with PFS < 6 months	[73]
**Other cancer types**								
Advanced thoracic carcinoma (*n* = 42, 32M/10F)	IV	PD-1 blockade	Assessment of predictive potential of the gut microbiome prior to ICI therapy	Feces	16S rRNA sequencing	↑Akkermansiaceae, Enterococcaceae, Enterobacteriaceae, Carnobacteriaceae, and Clostridiales Family XI in the R group, correlated with longer PFS	α-diversity; β-diversity; No significant differences between R and NR	[57]
Advanced-stage GI (*n* = 74, 53M/21F)	III/IV	Anti-PD-1 or anti-PD-1/anti-CTLA-4	Assessment of gut microbiota composition prior to and during immunotherapy, along with clinical evaluations	Feces	16S rRNA sequencing, MSS	↑Prevotellaceae, Ruminococcaceae, and Lachnospiraceae in R ↓Bacteroidaceae in R ↓*Prevotella/Bacteroides* ratio in R SCFA producers (*Eubacterium*, *Lactobacillus*, and *Streptococcus*) → positively associated with anti-PD-1/PD-L1 response	α-diversity; No significant differences between R and NR	[61]
Hepato-cellular carcinoma (*n* = 8)	BCLC Stage C	PD-1 blockade	Assessment of gut microbiota composition at baseline and during ICIs infusion	Feces	MSS	Higher taxa richness and more gene counts in R vs. NR ↑Proteobacteria in NR during therapy	Dissimilarity in β-diversity across patients	[62]

GM: Gut microbiota; CTLA-4: Cytotoxic T-lymphocyte antigen 4; F: Female; GI: Gastrointestinal; HC: Healthy controls; HMP: Human Microbiome Project; ICIs: Immune Checkpoint Inhibitors; M: Male; MSS: Metagenomic Shotgun Sequencing; ND: None Described; NSCLC: Non-Small Cell Lung Cancer Treatment; NR: Non Responders; OS: Overall Survival; PD-1: Programmed cell death protein 1; PFS: Progression-free survival; POR: Postoperative recurrence; R: Responders; spec: specimens; TTF: Time to treatment failure; UPLC-MS/MS: Unbiased Gut Metabolomic Profiling with Ultrahigh Performance Liquid Chromatography-Tandem Mass Spectroscopy.

Overall, research outcomes outline significant differences in microbial composition between responders and non-responders, often presenting conflicting results about the correlation of specific commensals with the clinical response of patients to ICIs treatment. These inconsistencies may be due to host-specific factors, including the unique microbiota of patients, differences in study design, and methods of microbiota analysis. However, as presented in a recent systematic review [74], the presence of *B. longum* and *F. prausnitzii* is correlated with better responses to ICIs. Additionally, studies collectively show that increased alpha-diversity may be associated with better responses to immunotherapy [12,59,75] and prolonged PFS [51,59,73]. Validation of these initial indications in larger cohorts and multi-center studies will greatly benefit the field. Towards this direction, three studies recruiting >450 patients aim to decipher the correlation of gut microbiome with ICI response and incidence of adverse effects in melanoma (NCT03643289), NSCLC, melanoma, and renal cell carcinoma (NCT04107168, NCT05037825), and triple-negative breast cancer patients (NCT05037825) (Table 2).

## 5. Modulation of Gut Microbiota and Efficacy of Cancer Immunotherapy

Understanding the intricacies of gut microbiota and decoding its delicate structure and its complex functions set the stage for its precise manipulation to elicit favorable outcomes on the health of the host. Indeed, as evidenced in ongoing clinical trials, the field is moving towards the design of strategies to manipulate its composition to maximize the efficacy and minimize the toxicity of ICI treatment (Table 3). Common methods for microbiome manipulation are dietary interventions, administration of antibiotics, probiotics, prebiotics or synbiotics, and fecal microbiota transplantation (FMT). Among these strategies, antibiotics can dramatically change the gut microbial landscape, and their use prior to or during immunotherapy may limit its efficacy against solid tumors [76,77]. Nevertheless, recent studies showed that antibiotics did not influence the response of patients with microsatellite instability, high (MSI-H)/deficient mismatch repair (dMMR) tumors [78], or NSCLC [79]. Evidently, more studies are required to determine the relationship of antibiotic consumption to ICI response.

Diet is a predominant modulator of the gut microbiome and, subsequently, of human health. Eating habits were criticized for their potential contribution to carcinogenesis. Most profoundly, the excessive intake of dietary protein from red meat and low fiber consumption were positively linked to CRC development [80]. On the other hand, polyphenols and foodstuffs with high fiber content are shown to promote antitumor immunity [81]. Shaping a diet based on the ability of foods to modulate the microbiome–immune axis is referred to as “immunonutrition” [82]. Microbial taxa can respond differentially to dietary components, ultimately leading to structural changes in the gut microbiota. In greater detail, a diet rich in meat protein and high-saturated fat supports the proliferation of *Bacteroides* ssp. and *Bilophila* spp, whereas both digestible carbohydrates and undigestible fiber induce the proliferation of lactobacilli and bifidobacteria while decreasing *Clostridium* populations [83]. These structural changes can be reflected in the metabolic profile of the gut micro-ecosystem. Indeed, SCFAs are mainly derived from the fermentation of undigestible fiber (or prebiotics) or of plant proteins of the Mediterranean diet by various species, including *A. muciniphila*, *Roseburia* spp., *B. longum*, and *F. prausnitzii* [84]. In a recent cohort study, melanoma patients treated with ICIs who reported sufficient fiber uptakes showed significantly longer PFS than patients reporting lower intakes [85]. A study aiming to decipher the diet–microbiome–immune system interplay in NSCLC patients receiving PD-1/PD-L1 blockade is currently ongoing (NCT04636775), whereas two interventional studies will explore the effect of fiber intake on the response of melanoma patients to immunotherapy as well as the incidence of adverse events and the quality of life of its participants (NCT04866810, NCT04645680). The ketogenic diet, a very low-carbohydrate diet rich in fat and proteins, although previously considered controversial, is shown today to have positive effects on diabetes prevention, obesity, and possibly neurological disorders [86]. In this context, the effects of this diet on the response rate of patients with metastatic renal cell carcinoma are being examined in an interventional, non-randomized study (NCT05119010). Last, dietary supplements with indications of immunomodulatory activity are also being tested in two studies recruiting patients with metastatic NSCLC (NCT04009122, NCT05384873).

The microbial efflux from fermented foodstuffs can significantly alter, even transiently, the gut micro-ecosystem [87]. Probiotics, beneficial bacteria that may elicit health benefits when administered in adequate amounts, are usually included as starter or non-starter cultures in dairy and non-dairy products. These bacteria can modulate the gut microbiota composition [88] or induce immunomodulatory effects through their surface molecules and excreted metabolites [89]. Ongoing interventional studies will determine the effect of mono- or multi-species probiotic supplementation on ICI response. The influence of *Clostridium butyricum* CBM 588, a commensal probiotic previously shown to elicit anti-tumor effects in murine models of bladder cancer [90], will be evaluated on microbiome and immune parameters of patients with renal cell carcinoma at various stages, those treated with PD-1 blockade and a small-molecule inhibitor of tyrosine kinases (cabozantinib) (NCT05122546), and on patients with advanced kidney cancer receiving anti-CTLA-4/anti-PD-1 combination therapy (NCT03829111). Similarly, the effect of *Lactobacillus rhamnosus* Probio-M9 on patients with liver cancer (NCT05032014), *L. Bifidobacterium* V9 (NCT05094167) or BiFico supplementation (NCT04699721) on patients with NSCLC, and *Bifidobacterium*, *Lactobacillus*, and *Enterococcus* administration on patients with urothelial bladder carcinoma (NCT05220124) undergoing ICI treatment will be measured using survival outcomes (e.g., PFS, overall survival (OS)), immunological, and gut microbiome markers (Table 3). In this frame, we are evaluating the effect of a multi-strain probiotic supplement on immunological parameters (T cell populations, production of pro-inflammatory cytokines), gut microbiota structure and function, quality of life, and response to ICI therapy in a multi-center study recruiting patients with advanced solid tumors who are naive to immunotherapy.

FMT is one of the most promising methods of microbiota manipulation, and it was recently approved by the FDA for the prevention of recurrent *Clostridium difficile*-induced colitis in adults [91]. During FMT, patients receive the fecal microbiome of donors orally or, less commonly, via colonoscopy or gastroscopy [92]. For cancer immunotherapy, transplants of defined consortia of bacteria or fecal matter derived from responders could find application in reversing resistance to ICIs. Indeed, recent clinical studies show that FMT from donors showing complete response to treatments augmented the effects of ICI in a subset of melanoma patients, showing promise for further investigation [93,94]. In this context, the ongoing MITRIC study aims to elucidate the effect of FMT from responders to patients with solid tumors who have initially failed immunotherapy by measuring the parameters of response to treatment and therapeutic outcomes (NCT05286294). In the case of FMTs, similar to diet interventions for the optimization of ICI therapeutic effects, ongoing clinical trials mainly target advanced-stage lung cancer (NCT05502913, NCT05008861, NCT04924374) or melanoma (NCT03772899, NCT03353402, NCT05251389), and a smaller portion of studies is aimed towards gastrointestinal cancers, including small intestinal or colorectal (NCT04729322) and renal cancer (NCT04758507, NCT04163289). The main outcomes in these studies are the tolerability and feasibility of FMT as well as the objective response of patients to treatment and the incidence of immunotherapy-related adverse effects. Alternative to FMT, bacterial consortium transplantation (BCT) is the administration of a well-defined bacterial mix derived from healthy donors. In this context, MET-4, a microbial ecosystem therapeutic, will be used in a randomized, open-label study recruiting patients with solid carcinomas who receive immunotherapy. This study will evaluate the safety and efficacy of the intervention, monitoring PFS, immune-related blood, and TME parameters (NCT03686202).

## 6. Incorporating Gut Microbiome Research in the Clinic—Pitfalls and Opportunities

**Figure 1:**
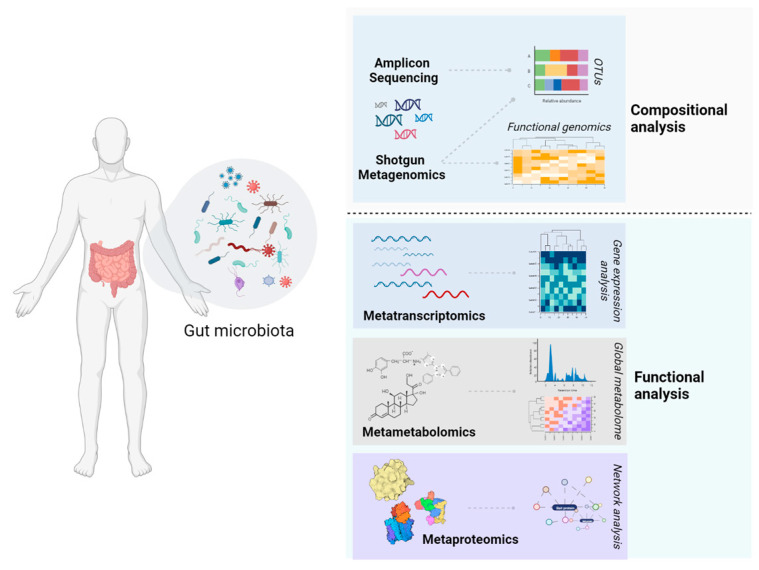
Omic platforms for the characterization of gut microbiota. Amplicon sequencing or metataxonomics detect only part of the gut microbiota, whereas shotgun metagenomics targets all organisms (bacteria, viruses, fungi, and protozoa) present in the community. The study of the transcripts (metatranscriptomics), metabolites (metametabolomics), or proteins (metaproteomics) facilitates the construction of microbe–microbe and microbe–host interaction networks. Created with BioRender.com.

Today, the microbiome field has experienced many breakthroughs supported by state-of-the-art platforms aiming to crack the microbial code. However, the implementation of the accumulated knowledge in the clinic to improve ICI efficiency is lagging. Host-associated microbial communities comprise a complex micro-ecosystem that presents great variability among hosts and physiological states. This remarkable complexity remains a limiting factor for the comprehensive characterization of microbiome structure and function in health and disease as well as for its application in the clinical setting. In this sense, a consensus “core” microbiome composition is yet to be defined. Likewise, although the term “dysbiosis” is used ad hoc to describe deviations from the healthy gut microbiome, no solid definition has been given in the literature [95]. Sex, age, body-to-mass index (BMI), alcohol consumption frequency, bowel movement quality, antibiotic, prebiotic, probiotic, or synbiotic consumption, as well as genetic parameters and disease progression are common contributors to this profound heterogeneity [96]. Thus, the composition of non-homeostatic gut microbiota may be a person- rather than a disease-specific matter. However, it was suggested that the functional capacity of this community remains relatively stable over time, and thus, characterizing genes rather than microbe content could be a more efficient way to determine baseline or dysbiotic microbiota [97]. Large-scale analysis of the gene expression and biosynthetic capacity of the gut microbial community can be performed using metatranscriptomics, metaproteomics, and metabolomics (Figure 1). Although technological limitations still apply for these platforms [98,99], their integration into human microbiome studies offers clear advantages, providing better insights into dysbiosis and host-microbiome signaling [100]. In this frame, ongoing studies incorporate metabolomics into their design to determine the functional changes accompanying differential responses to ICI therapy (NCT03643289, NCT05251389, NCT03772899, NCT05199649) (Table 2 and Table 3). Importantly, the IRIS study aims to tackle resistance to immunotherapy by incorporating multi-omic approaches. More specifically, the genomic, transcriptomic, metagenomic, and epigenetic signatures of resistance will be evaluated in a cohort of patients with solid tumors (NCT04243720).

For omic platforms to accurately reveal the structure and function of microbial communities, choosing the appropriate types of sampling, collection, and handling methods is of imperative importance. In that sense, most studies investigating the gut microbiome–host axis rely on stool samples; however, the composition of the stool could differ from that of the intestinal microbiota [101]. Of note, variations in the community structure were recorded longitudinally and transversally, with differences in microbial abundance at the length of the GI tract and from lumen to mucosa [102]. Protocols for microbiome sample handling are established for stools [103]. However, no consensus exists for the collection, handling, and manipulation of lung, tumor, skin, or urogenital tissues, making the proper interpretation of results challenging. Indeed, a recent groundbreaking perspective suggested that the fetal microbiome may be an artifact derived from sampling or handling contamination [104]. Understanding the contribution of extraintestinal and intratumor microbiota on carcinogenesis could provide new opportunities for the development of novel diagnostic or therapeutic modalities. To this end, the widespread application of metagenomics revealed the presence of distinct microbial communities residing on the skin, lung, mouth, and genitourinary tract that can elicit systemic effects on the host [105].

Although most of the published and ongoing studies focus on the bacterial component of the gut microbiota, the wealth of fungi, viruses, archaea, and protists residing in the cavities and surfaces of the host is often overlooked. Recent studies indicate that these communities play an important biological role in the health of the host [106,107]. Shotgun metagenomics is an untargeted and unbiased strategy that can offer a holistic picture of microbial communities at the time of sampling. It can simultaneously detect rare bacterial taxa, fungi, viruses, bacteriophages, and protists and shed unprecedented light into the microbial “dark matter”. Importantly, it can be supplemented by functional genomics analysis to pinpoint novel genes for bacteria–host interactions as well as for other phenotypes of interest, such as antimicrobial resistance [108]. On the contrary, amplicon sequencing (or metataxonomics), which is most often used in clinical studies, can only determine the presence of bacteria or fungi, depending on experimental design. Thus, the use of shotgun metagenomics rather than amplicon sequencing could facilitate the profiling of the intestinal and extraintestinal microbiota of patients at greater depth (Figure 1).

The design of strategies for microbiome manipulation has flourished lately. From diet interventions to the consumption of microbial matrices of fermented products, FMTs or BCTs, mono- or multi-strain probiotic supplements, this field elicited scientific interest [109]. However, interventional studies may be prone to several pitfalls. First, the microbial composition of fermented foods, probiotic supplements, or FMTs are not reported in great detail. Most commonly, these bacteria are not described in adequate taxonomic depth and the strain names are often not disclosed; thus, this hinders the application of the findings in the clinic and significantly undermines the efforts of replication studies and metanalyses. Recent studies underscore the fact that probiotics possess strain-, host-, and disease-specific activity [110]. More specifically, members of the same species were observed to either induce pro- or anti-inflammatory activity or alter the gut microbial communities of the host with different capacities [111]. Indeed, two elegant studies showed that probiotics present a host-specific pattern of colonization, being able to adhere to the mucosa of the host with variable efficiency [88,112]. The administration of viable bacteria could elicit concerns about the safety of frail individuals, including cancer patients. Probiotics possess the “generally recognized as safe” (GRAS) FDA status, and thus, they are safe for consumption. However, some LAB strains may exhibit hemolytic activity or carry transferable antibiotic-resistance genes. Toward this direction, the introduction of genomics in probiotic research provided powerful prediction tools to identify genes involved in the pathogenic phenotypes using the assembled whole genome sequence (WGS) before their subsequent validation in vitro and in vivo [113]. In this context, we recently published the WGS of three lactobacilli of probiotic and biotechnological interest and examined the genetic bases of these characteristics by employing comparative genomics and annotation algorithms [114,115,116]. Alternative to the administration of whole bacterial cultures, recent studies focused on the characterization of the health-promoting properties of metabolites and the excreted or cell surface proteins of lactobacilli. Concomitantly, studies of mice showed promising results on the effect of bacterial metabolites on response to ICIs [117,118], granting further research.

Although generally regarded as safe, FMT could rarely transmit enteropathogens, bacteriophages, and resistant bacteria to the recipient, which could ultimately lead to infectious disease and even death [119]. Recent studies additionally showed that the gut microbial composition may carry a “signature of disease”. Indeed, it was reported that mice that received patient feces developed similar manifestations of disease to human donors [120]. These findings suggested that caution should be taken in the application of FMT in the clinic, as the long-term effects of this intervention have not been adequately reported. This highlights the need to screen the health status of the donor based on hereditary and spontaneous diseases and lifestyle. In this context, the complete profiling of the microbial samples to be transplanted could alleviate these issues [121]. BCTs could be a viable alternative, as the bacterial component of the mix is clearly defined, whereas the use of gut commensals, including *A. muciniphila* or their metabolites, could be better tolerated by the host [122].

In clinical microbiome research, carefully accounting for confounding factors is of profound importance, as the gut microbiome can be readily influenced by a plethora of genetic and environmental factors, as previously mentioned. In this context, the diet of participants in these studies should be closely monitored, even if the diet–microbiome-response to immunotherapy correlation is not a primary or secondary outcome of the study. Currently, most published studies do not take into consideration the diet of the participants, as no data for the habits of the participants exist, including their fiber and protein consumption as well as their use of prebiotic or probiotic supplements, prior to or during the study. Additionally, most clinical research is still conducted by recruiting white male participants; thus, the effect of gender and ethnicity on microbiome composition and, therefore, the response to ICIs is ignored (Table 1). To counteract this, an ongoing clinical study is incorporating the gender dimension in immunotherapy-related adverse effects by monitoring their incidence and correlation with immune-related markers and gut microbiota composition (NCT04435964) (Table 2). Furthermore, open access to anonymized raw data derived from the studies, including confounding factors, dietary habits, baseline microbiota composition, changes in disease- and ICI-relevant biomarkers, and clinical outcomes will facilitate metanalyses, and tremendously invigorate the field. To date, two metanalyses have been published on the correlation of gut microbiota with ICI response in melanoma patients based on clinical studies performing amplicon sequencing [123] or shotgun metagenomics [124] using a small sample of three or four studies, respectively. In this context, a metanalysis of a larger sum of studies with statistical tests that will account for confounding factors could be pivotal in bridging research data with clinical outcomes, paving the way for personalized ICI therapy.

## 7. Conclusions

The notion that the gut microbiome is a static observer was debunked by numerous studies showing its influence on host homeostasis and pathophysiology. Among its described functions, the gut microbiome plays an important role in the maturation and modulation of immunity in developing and adult hosts. Thus far, several studies have indicated that specific gut populations could be correlated with responses to immunotherapy. Concomitantly, interventional methods for gut microbiota manipulation to support the proliferation of beneficial taxa while suppressing bacteria related to poor clinical outcomes are strategies that should be further investigated. Although there is a long road ahead, concentrated efforts with well-designed clinical studies that minimize the effect of confounders and appropriate metanalyses could streamline the incorporation of microbiota-targeting drugs for beneficial outcomes in clinical practice.

## Figures and Tables

**Table 2:** Ongoing observational clinical trials investigating the effect of gut microbiota composition on ICI immunotherapy efficacy and therapeutic toxicity.

NCT Number	Cancer Type—Disease Stage	Sample Size	Immunotherapy Type	Samples	Purpose—Expected Findings
NCT04136470	NSCLC Melanoma	130	ICIs (anti-PD-1, anti-PD-L1 or anti-CTLA-4)	Feces, Blood, Biopsy	Detection of differences in GM between ICI responders and non-responders.
NCT04957511	Gynecologic (advanced or recurrent)	30	ICIs (Not specified)	Feces, Blood, Saliva, Vaginal swab	Inter- and intra-patient microbiome changes related to immunotherapy. Association with the response to treatment.
NCT04636775	NSCLC (I-IV)	46	PD-1 blockade	Feces, Nasal and Buccal swabs	Association between GM and prediction of the effectiveness of immunotherapy treatment.
NCT03643289	Melanoma (III/IV)	450	ICIs (Not specified)	Feces, Blood	Assessment of the impact of the GM on treatment response rates and side effects induced by immunotherapy.
NCT04107168	Melanoma, Renal, Lung (III/IV)	1800	ICIs (anti-PD-1, anti-PD-L1 or anti-CTLA-4)	Feces, Saliva	GM correlations with efficacy and toxicity of ICIs in patients with advanced cancer.
NCT05037825	NSCLC, Melanoma, RCC, Triple-Negative Breast	800	ICIs (anti-PD-1, anti-PD-L1 or anti-CTLA-4)	Feces, Blood	Associations between the gut microbiota (composition and function), host immune system, and ICI treatment efficacy.
NCT04954885	Lung (III-IV), NSCLC (IV)	150	ICIs (PD-1 blockade)	Feces	Estimation of the extent to which future interventions that seek to rationally modify the gut microbiome and/or functional status can improve outcomes.
NCT04579978	Advanced solid tumor	60	ICIs (Not specified)	Feces, Blood	Characterization of the diversity of gut bacteria and assessment of the potential mechanisms by which gut bacteria impact the immune response.
NCT04435964	Melanoma, Lung, Head and Neck, Urogenital Neoplasms, Breast	100	ICIs (Not specified)	Feces, Blood	Investigation of sex differences in irAEs in relation to clinical factors and genetic, immunological, and hormonal profiles.
NCT04243720	Solid tumors, Metastatic cancers	100	Not specified	Feces, Blood, Tumor sample	Investigation of resistance to immunotherapy and its correlation with different genomic, transcriptomic, immunophenotypic, and/or epigenetic profiles.
NCT04204434	Advanced-stage cancer	150	ICIs (Not specified)	Feces, Tissue, Blood, Plasma	Characterization of serum and microbial predictors of response to response and toxicity.
NCT04913311	NSCLC	150	ICIs (Not specified) and chemotherapy	Feces, Blood, Saliva	Creation of database by correlating blood, stool and saliva biomarkers, and data from lung function tests with treatment outcomes and side effects.

CRC: Colorectal Cancer; CTLA-4: Cytotoxic T-lymphocyte antigen 4; GM: Gut Microbiota; ICIs: Immune Checkpoint Inhibitors; irAEs: Immune-Related Adverse Events; NSCLC: Non-Small Cell Lung Cancer; PD-1: Programmed cell death protein 1; RCC: Renal Cell Carcinoma.

**Table 3:** Ongoing interventional clinical trials on intestinal microbiota and anti-tumor immunotherapy.

NCT Number	Status	Cancer Type (Disease Stage)	Sample Size	Immunotherapy Type	Intervention	Purpose—Outcomes	Type of Study	Phase
NCT04645680	Recruiting	Cutaneous melanoma (III–IV), MM, UM	42	PD-1 blockade	Dietary intervention	Changes in systemic and tumor immunity, microbiome, and metabolic profile of patients, QoL, symptom profile, incidence of AE	Randomized, parallel assignment, double blind	Phase II
NCT04866810	Recruiting	UM	80	Anti-PD-1/PD-L1 monotherapy	Dietary intervention	PFS, QoL, ORR	Randomized, parallel assignment, open label	N/A
NCT05384873	Not yet recruiting	NSCLC	180	Not specified	Dietary intervention	PFS, QoL, DoR, incidence of AE, physical activity level	Randomized, parallel assignment, open label	N/A
NCT04636775	Recruiting	NSCLC (IV), recurrent NSCLC	46	PD-1/PD-L1 blockade	Observational	Correlation of gut microbiota with response, adverse effects incidence, tumor tissue PD-L1 expression and diet	Observational, cohort prospective study	N/A
NCT05083416	Recruiting	Head and neck	62	Not specified	Dietary intervention (Fasting)	Compliance, correlation of gut microbiome and microbial metabolites	Non-randomized, parallel assignment, open label	N/A
NCT04009122	Active, not recruiting	NSCLC	206	Not specified	Dietary intervention	QoL, changes in microbiota, interleukin, and cytokine levels	Randomized, parallel assignment, quadruple masking	N/A
NCT05119010	Not yet recruiting	Metastatic RCC (IV)	60	Anti-PD-1/anti-CTLA-4 combinatory treatment	Dietary intervention	QoL, OS, ORR, safety assessment, PFS	Non-randomized, parallel assignment, open label	N/A
NCT05032014	Recruiting	Liver	46	PD-1 blockade	Dietary intervention *L. rhamnosus* Probio-M9	Objective remission rate, PFS, OS	Randomized, parallel assignment, quadruple masking	N/A
NCT05094167	Recruiting	NSCLC	46	PD-1 blockade	Dietary intervention *L. Bifidobacterium* V9 (Kex02)	Objective remission rate, PFS, OS	Randomized, parallel assignment, quadruple masking	N/A
NCT04699721	Recruiting	NSCLC (III)	40	PD-1 blockade and chemotherapy	Dietary intervention—BiFico powder	Adverse effects, ORR, DFS, OS	Single group assignment, open label	Phase I
NCT03829111	Active, not recruiting	RCC (III–IV), Unresectable RCC	30	Anti-PD-1/anti-CTLA-4	Dietary intervention—*Clostridium butyricum* CBM 588	OS, PFS, change in feces bifidobacterial count, change in Shannon index	Randomized, parallel assignment, open label	Phase I
NCT05220124	Recruiting	Bladder Urothelial Carcinoma	190	Not specified	Dietary intervention *Bifidobacterium*, *Lactobacillus* and *Enterococcus* capsules	PFS, DoR, OS, ORR, SAE	Randomized, parallel assignment, open label	Phase IV
NCT05122546	Recruiting	RCC (III–IV), Unresectable RCC, Metastatic RCC	30	Not specified	Dietary intervention—*Clostridium butyricum* CBM 588	OS, PFS, change in feces bifidobacterial count, change in Shannon index, immunomodulation	Randomized, parallel assignment, open label	Phase I
NCT04163289	Recruiting	RCC (III–IV)	20	PD-1 blockade	FMT Donors: HC	Safety of FMT combination treatment Changes in the immune populations, microbiome profile of patients, response to treatment, and OS	Single group, open Label	Phase I
NCT04264975	Recruiting	Solid Carcinoma	60	Not specified	FMT (via colonoscopy) Donors: Patients with CR or PR	Prospects of utilization of microbiome as biomarkers and therapeutics in immuno-oncology	Single group, open label	N/A
NCT03353402	Unknown	MM (IV) Unresectable Melanoma (III)	40	Not specified	FMT (via colonoscopy and oral capsules) Donors: Patients with MM who responded to immuno-therapy	Safety of FMT treatment. Changes in the composition and activity of immune populations, response to treatment.	Single group, open label	Phase I
NCT04521075	Recruiting	MM (IV) Unresectable Melanoma (III) NSCLC (IV)	42	PD-1 blockade	FMT (oral capsules) Donors: Patients with DR, CR	FMT-related AE, ORR, PFS, OS, DoR, irAEs, immune activation markers	Single group, open label	Phase I and II
NCT04577729	Recruiting	Melanoma (III–IV)	60	Not specified	FMT (oral capsules) Fecal implant donors; Prior malignant melanoma patients in remission for at least 1 year after ICIs	PFS, gut microbiota analysis, adverse effects, neutrophil-to-lymphocyte ratio	Randomized parallel assignment, double blind	N/A
NCT03341143	Active, not recruiting	Melanoma	18	PD-1 blockade	FMT (via colonoscopy) Donors: Patients treated with a PD-1 inhibitor, rendered disease-free as a result	ORR, OS, immune parameters, frequency of grade III/IV toxicities	Single group, open label	Phase I
NCT03772899	Active, not recruiting	Melanoma (advanced stage)	20	PD-1 blockade	FMT (oral capsules) Donors: HC	Safety assessment, ORR	Single group, open label	Phase I
NCT04116775	Recruiting	mCRPC	32	PD-1 blockade	FMT (via endoscopy) Donors: Patients who respond to treatment at an earlier stage	Anticancer effect of FMT	Single group, open label	Phase II
NCT04988841	Recruiting	Unresectable or MM (III/IV)	60	ICIs (anti-PD-1 or anti-CTLA-4)	FMT-pooled donor	Assessment of the tolerance and clinical benefit of FMT	Randomized parallel assignment, double blind	Phase II
NCT04951583	Recruiting	NSCLC, Advanced Melanoma (IV)	82	PD-1 blockade	FMT (Investigational capsules)	Assessment of the impact of FMT on ICI response and survival Evaluation of the changes in patient’s GM composition and tumor microenvironment contexture following the combination treatment of ICI and FMT	Single group, open label	Phase II
NCT05502913	Not yet recruiting	Metastatic Lung Cancer	80	PD-1 blockade	FMT (Oral capsules) Donors: CR	PFS, OS, ORR, microbiome analysis, safety, feasibility, immunomodulation	Randomized, parallel assignment, quadruple masking	Phase II
NCT05286294	Recruiting	Melanoma (IV), Head and Neck Squamous Cell Carcinoma, Cutaneous Squamous Cell Carcinoma, Clear Cell Renal Cell Carcinoma	20	Not specified	FMT (Oral capsules) Donors: ICI R	PFS, OS, ORR, microbiome analysis, safety, feasibility, immunomodulation, QoL	Single group, open label	Phase II
NCT05008861	Not yet recruiting	Advanced or Metastatic NSCLC	20	PD-1/PD-L1 blockade	FMT (Oral capsules)	ORR, microbiome analysis, safety, FMT-related adverse effects or treatment-related adverse effects, immunomodulation	Single group, open label	Phase I
NCT05251389	Recruiting	Melanoma (III–IV)	24	Not specified	FMT from responders or non-responders to ICI treatment	Efficacy (SD, PR, CR), microbiome analysis, safety, immunomodulation, changes in metabolome	Randomized, parallel assignment, quadruple masking	Phase I/II
NCT04924374	Recruiting	NSCLC (III–IV)	20	PD-1 blockade	FMT (Oral capsules) Pooled fecal microbiota capsules from 1 donor based on composition	Safety and efficacy (iRECIST criteria)	Randomized, parallel assignment, open label	N/A
NCT04729322	Recruiting	CRC (IV), Metastatic CRC, Small intestinal adenocarcinoma (IV), Metastatic small intestinal adenocarcinoma	15	PD-1 blockade	FMT (via colonoscopy) Donors: PD-1 responding CRC patients	ORR	Non-randomized, parallel assignment, open label	Phase II
NCT04758507	Recruiting	RCC	50	Not specified	FMT (via colonoscopy and frozen capsules) Donors: ICI R	PFS, PR or CR, OS, AE, gut microbiota diversity	Randomized, parallel assignment, quadruple masking	Phase I/II
NCT03686202	Active, not recruiting	Any Solid Tumor	65	PD-1/PD-L1 and/or anti-CTLA4 blockade	Microbial Ecocystem Therapeutics (MET, Oral administration) Donor: HC	Immunotherapy response, bacterial taxonomic diversity	Randomized, single group—open label	Phase II/III
NCT05273255	Recruiting	Any Solid Tumor (IV)	30	Not specified	FMT (Colonoscopic)—Donors: ICI R with stage III or IV solid cancers	PFS, OS, ORR, AE, QoL, gut microbiome profiling, immunomodulation	Single group—open label	N/A

AE: Adverse Effects; ICI: Checkpoint Inhibitor; CR: Complete Response; CTLA-4: Cytotoxic T-lymphocyte antigen 4; DFS: Disease Free Survival; DR: Durable response; DoR: Duration of Response; FMT: Fecal Microbial Transplantation; GM: Gut Microbiota; HC: Healthy controls; ICIs: Immune Checkpoint Inhibitors; irAEs: Immune-Related Adverse Events; mCRPC: Metastatic castration resistant prostate cancer; MET: Microbial Ecosystem Therapeutics; MM: Metastatic Melanoma; N/A: Not applicable; NSCLC: Non-Small Cell Lung Cancer; ORR; Overall Response Rate; OS: Overall Survival; PD-1: Programmed cell death protein 1; PFS: Progression-free survival; PR: Partial Response; QoL: Quality of Life; RCC: Renal Cell Carcinoma; SAE: Serious Adverse Effects; SD: Stable Disease; UM: Unresectable Melanoma.

## Data Availability

The data can be shared up on request.
